# Sexuelle Selbstbestimmung und Behinderung: 10 Jahre Projekt ReWiKs – Dynamiken und Erkenntnisse

**DOI:** 10.1007/s00103-026-04263-1

**Published:** 2026-06-26

**Authors:** Sven Jennessen, Tim Krüger

**Affiliations:** Institut für Rehabilitationswissenschaften, Humboldt-Universität zu Berlin, Unter den Linden 6, 10999 Berlin, Deutschland

**Keywords:** Sexualität, Eingliederungshilfe, Behinderung, ReWiKs, Sexuality, Integration assistance, Disability, ReWiKs

## Abstract

Sexuelle Selbstbestimmung von Menschen mit Behinderung ist weiterhin ein marginalisiertes Themenfeld im Kontext des Teilhabediskurses. Narrative und Fakten, die die (sexuellen) Lebenswirklichkeiten der Zielgruppe bestimmen, zeigen deutlich, dass die Realisierung sexueller Selbstbestimmung primär von behindernden gesellschaftlichen Vorstellungen, strukturellen Herausforderungen und paternalistischen Alltagspraktiken geprägt ist. Diese stehen zumeist im Widerspruch zu sexuellen Menschenrechten. Das Forschungsprojekt ReWiKs hat ein umfangreiches themenspezifisches Medienpaket entwickelt, Fortbildungen für Fachkräfte durchgeführt und partizipationsorientierte Gesprächsformate (Freiraum-Gruppen) für Menschen mit Behinderung konzipiert. Die empirischen Resultate dieser Aktivitäten zeigen bedeutsame Kompetenzentwicklungen der Fachkräfte, jedoch auch erhebliche strukturell und institutionell bedingte Herausforderungen in der Entwicklung und Umsetzung sexualfreundlicher Einrichtungskulturen. Die partizipativ konzipierten Gesprächsformate für Menschen mit Behinderung eröffnen Räume für Resonanz, Austausch, Anerkennung und sexuelle Bildung. Aus den Erkenntnissen des Projekts werden multidimensionale Konsequenzen für Teilhabefragen im Kontext selbstbestimmter Sexualität abgeleitet.

## Abstract (en)

Sexual self-determination for people with disabilities remains a marginalised topic in the context of the discourse on participation. Narratives and facts that define the (sexual) realities of life for the target group clearly show that the realisation of sexual self-determination is primarily shaped by disabling social attitudes, structural challenges and paternalistic everyday practices. These are mostly at odds with sexual human rights. The ReWiKs research project has developed a comprehensive topic-specific media package, conducted training courses for professionals and designed participation-oriented discussion formats (Freiraum-Gruppen) for people with disabilities. The empirical findings from these activities indicate significant competence developments among professionals, while also revealing considerable structural and institutional challenges in the development and implementation of sex-positive institutional cultures. The participatory discussion formats for people with disabilities create spaces for resonance, exchange, recognition and sexual education. The findings of the project have multidimensional consequences for participation issues in the context of self-determined sexuality.

## Einleitung

Die Realisierung sexueller Selbstbestimmung als sexuelles Menschenrecht [1] und Teil individueller Lebensführung wird im Teilhabediskurs nach wie vor nur marginal berücksichtigt (siehe auch Themenheft des *Bundesgesundheitsblatts* 9/2016 [2]). Dies überrascht insofern, da die Behindertenrechtskonvention der Vereinten Nationen (UN-BRK) in Artikel 23 unmissverständlich formuliert, dass die Vertragsstaaten sich gegen die Diskriminierung von Menschen mit Behinderung „in allen Fragen, die Ehe, Familie, Elternschaft und Partnerschaften betreffen“ [3], einsetzen und diesbezüglich konkret formulierte Zielsetzungen umsetzen sollen. Auf der Grundlage eines multidimensionalen Verständnisses von Sexualität, das den vielschichtigen thematischen Komplex von Liebe, Partnerschaft, Familie, Körperlichkeit und Nähe sowie Gender und Sex in seiner diversen Verfasstheit in den Blick nimmt [4], sind diese Aspekte als zumindest mittelbare Bestandteile sexueller Selbstbestimmung zu verstehen.

Dennoch wird das Thema Sexualität in der UN-BRK – als Ergebnis eines komplexen Aushandlungsprozesses [5] – nicht explizit genannt und sexuelle Fragen schwingen in den o. g. Themen nur implizit mit. Dies steht im Kontrast zu einem Verständnis von Sexualität als jeden Menschen und die menschliche Biografie umfassende „Lebensenergie“ [6], die in verschiedenen Lebensbereichen bedeutsam ist. Die Diskrepanz zwischen der individuellen und gesellschaftlichen Bedeutung von Sexualität einerseits und ihrer Marginalisierung in der UN-BRK andererseits spiegelt sich in der weiterhin randständigen Berücksichtigung sexueller Aspekte im Teilhabediskurs wider, die bislang nur punktuell überwunden wird. So ist als positiv anzusehen, dass in der 2. Welle des Teilhabesurveys nun explizit anerkannt wird, dass „Partnerschaft und Familie … zentrale Lebensbereiche (sind), die maßgeblich zur persönlichen Entfaltung und sozialen Teilhabe beitragen“ [7]. Entsprechend wurde dieses Themenfeld in die qualitative Vertiefungsstudie aufgenommen, aus der nun Erkenntnisse zu subjektiven Wahrnehmungen und Lebensrealitäten der Zielgruppe vorliegen.

Ein weiteres, politisch initiiertes und wissenschaftlich eng an den Lebensrealitäten von Menschen mit Behinderung ausgerichtetes Vorhaben ist das Forschungsprojekt „ReWiKs“ (*R*eflexion, *W*issen, *K*önnen – *s*exuelle Selbstbestimmung von Menschen mit Behinderungen), das unter Beteiligung von Akteur*innen der Eingliederungshilfe (EGH) und der Selbstvertretung umgesetzt wurde. Es wurde 2014 von der Bundeszentrale für gesundheitliche Aufklärung (heute Bundesinstitut für öffentliche Gesundheit, BIÖG) gefördert und zielte darauf ab, durch die Entwicklung themenspezifischer Materialien [8] zur Erweiterung der sexuellen Selbstbestimmung der Zielgruppe beizutragen.

Der Beitrag präsentiert auf Basis grundlegender und aktueller Erkenntnisse zu sexueller Selbstbestimmung die Aktivitäten des Projekts ReWiKs, seine Dynamiken und empirischen Erkenntnisse. Eine abschließende kritische Analyse zeigt weitere Entwicklungsschritte auf, um die Thematik als relevanten Aspekt in Theorie und Praxis der Teilhabe von Menschen mit Behinderung zu verorten.

## Sexuelle Fremdbestimmung: Realitäten und Narrative

Den einleitend bereits angedeuteten ableistischen, negierenden und bagatellisierenden Positionen zu Fragen der Sexualität von Menschen mit Behinderung liegen hartnäckig persistierende Mythen zugrunde. Von Ortland [6] komprimiert präsentiert zeigen sich diese z. B. in der Vorstellung, dass als Unattraktivität gelesene körperliche Normabweichung vor sexueller Gewalt schütze oder dass das Sexualverhalten von Menschen mit Lernbeeinträchtigungen triebhaft sei. Auch Vorstellungen von Asexualität – vor allem bei Menschen mit komplexer Behinderung –, eine fehlende Wahrnehmung sexueller Gewalt aufgrund kognitiver Beeinträchtigungen sowie sexuell distanzloses Verhalten, das sexuelle Übergriffe geradezu provoziere, finden sich als Narrative in der Betrachtung der Sexualität der Zielgruppe. Wenngleich es für diese Vorstellungen keinerlei empirischen Belege gibt, sind sie immer noch Bestandteil des Diskurses. In der Verantwortung von Akteur*innen aus Wissenschaft und Praxis liegt es demnach, diese nicht nur infrage zu stellen, sondern ihrer kontinuierlichen Reproduktion empirische Belege entgegenzusetzen.

Hierzu ist in den vergangenen Jahren eine überschaubare Anzahl an Studien entstanden, die Teilbereiche des Themas beleuchten und fundieren. Diese weisen übereinstimmend darauf hin, dass es primär Umwelt- und Lebensbedingungen sind, die die sexuelle Selbstbestimmung behindern und individuelle Beeinträchtigungen meist erst in der Wechselwirkung mit Kontextfaktoren eine die Sexualität behindernde Wirkung entfalten. So finden sich in Einrichtungen der EGH häufig aktive oder passive Widerstände [9] gegen Weiterentwicklungen zu sexualfreundlichen Organisationen, die ernst genommen und kommuniziert werden müssen, um Entwicklung zu ermöglichen.

Um die Lebenslagen von Menschen mit Behinderung in ihrer Bedeutung für Fragen sexueller Selbstbestimmung nachvollziehen zu können, werden daher im Folgenden einige Fakten dargestellt, die von unmittelbarer Relevanz für die Thematik sind.

So teilen sich laut der 1. Repräsentativbefragung zur Teilhabe von Menschen mit Behinderungen [10] 15 % der Bewohner*innen von Einrichtungen der EGH ein Badezimmer, nur 67 % der Befragten mit eigenem Bad können dieses abschließen. Immer noch 12 % der Menschen in gemeinschaftlichen Wohnformen leben dauerhaft in einem Doppelzimmer. Zurecht werden diese Wohnformen auch als „unfreiwillig hergestellte Gemeinschaften“ bezeichnet, da in diesen die Wunsch- und Wahlmöglichkeiten des alltäglichen Lebens eingeschränkt sind [11]. Vor dem Hintergrund dieser Rahmenbedingungen wurden in den vergangenen Jahren Ansätze zur Etablierung sexualfreundlicher Wohneinrichtungen in der EGH formuliert [12–15]. Dass trotz erreichter Fortschritte die Realisierung des Rechts auf sexuelle Selbstbestimmung von Menschen mit Behinderungen dort immer noch aussteht [16], ist multifaktoriell bedingt. Neben Unsicherheiten und unzureichenden sexualpädagogischen Kompetenzen der Fachkräfte, den strukturellen Gegebenheiten und der marginalen Verankerung des Themas in Leitbildern, Konzepten und Prozessen der Organisationsentwicklung spielt auch die doppelte Tabuisierung von Sexualität und Behinderung eine bedeutsame Rolle.

All dies prägt die sexualbiografischen Erfahrungen und die Möglichkeiten der Realisierung sexueller Selbstbestimmung von Menschen mit Behinderung. Nachfolgend wird eine Auswahl zentraler Einflussfaktoren skizziert, die im Zusammenhang mit einer selbstbestimmten Sexualität stehen:

- *Viel Fremdbestimmung – wenig (sexuelle) Selbstbestimmung*: Die skizzierten Bedingungen in institutionellen Wohnformen treffen häufig auf frühe Erfahrungen von Fremdbestimmung, Bevormundung und daraus resultierendem mangelnden Zutrauen behinderter Menschen in eine selbstbestimmte Lebensführung. Bei Menschen mit körperlichen Beeinträchtigungen kann das Körpererleben zudem durch die schon früh begonnene und häufig lebenslang erfahrene Enteignung des eigenen Körpers beeinflusst sein, wodurch der Umgang mit Scham‑, Intim- und Care-Grenzen tangiert ist [17].
- *Geringe Beachtung und Realisierung sexueller und reproduktiver Rechte: *Zinsmeister [18] stellt zu reproduktiven Rechten fest, dass die Realität in Deutschland für Menschen mit Behinderung noch weit davon entfernt ist, den rechtlichen Vorgaben zu entsprechen. So würden die sozialen und sexuellen Kontakte von erwachsenen Bewohner*innen in besonderen Wohnformen noch häufig reguliert (z. B. bzgl. Übernachtungsbesuchen) und/oder an die Einnahme von Verhütungsmitteln gekoppelt. Der Einsatz von Depotpräparaten zur Empfängnisverhütung und eine überdurchschnittlich häufige Rate an Sterilisationen sind zudem Bestandteil der Lebensrealitäten von Frauen in besonderen Wohnformen [18].
  Juristisch bedeutsam ist zudem, dass gesetzliche Betreuer*innen verpflichtet sind, die erwachsenen Bürger*innen bei ihrer Entscheidungsfindung in sexuellen Fragen zu unterstützen und bis auf wenige gravierende und gesetzlich geregelte Ausnahmen in ihrem Handeln *ausschließlich* an die Wünsche der betreuten Person gebunden sind.
- *Stark heteronormativ geprägte Lebenswelten*: Menschen mit Behinderung erleben in ihren unmittelbaren Lebenswelten selten queere Zugangsweisen zu sexuellen Themen. Verschiedene internationale, aber auch vereinzelte qualitative Studien aus Deutschland [19] zeigen, dass es Menschen mit Lernschwierigkeiten schwerfällt, sich gegenüber dem sozialen Umfeld zu outen („Secret Loves, Hidden Lives“ [20]). Es lässt sich ein großer Bedarf feststellen, Einrichtungen der EGH, aber auch die queeren Communitys für die Lebenslagen *queerer Menschen mit Behinderung* und gegenseitige Ausgrenzungsmechanismen zu sensibilisieren [21].
- *Seltenere langjährige Partnerschaftserfahrung und Elternerfahrungen*: Menschen mit Behinderung haben seltener langjährige Partnerschaften und ihre Partner*innen haben überdurchschnittlich häufig ebenfalls eine Beeinträchtigung. Dies kann das gegenseitige Verständnis fördern, aber auch zusätzliche Belastungen durch gemeinsame Herausforderungen hervorbringen [7].
  Schwierig gestaltet sich die Realisierung von Kinderwünschen bei Menschen mit Lernbeeinträchtigungen – trotz des Rechts auf begleitete Elternschaft [22, 23]. Dies korrespondiert mit den genannten Einschränkungen der Realisierung reproduktiver Rechte, reduzierten Kontaktmöglichkeiten und gesellschaftlich konstruierten Bildern von Menschen mit Beeinträchtigungen als „hilfsbedürftige“ Personen. Selbst Fachkräfte, die mit Menschen mit Lernschwierigkeiten arbeiten, stimmen nur zu 31 % zu, dass diese auch Kinder bekommen sollten, wenn sie dies möchten [24].
- *Eingeschränkte Möglichkeiten der Kontaktaufnahme (real und digital):* Assistenznehmer*innen haben oft eingeschränkte Möglichkeiten, Sexual- oder Liebespartner*innen kennenzulernen. Freizeitaktivitäten beschränken sich oft auf andere Menschen mit Behinderung oder exklusive Sozialräume. Barrieren bei der digitalen Teilhabe [25] erschweren zusätzlich das Kennenlernen. Menschen mit Behinderung geben häufiger an, keinen engen Freundes- oder Bekanntenkreis zu haben, verbringen deutlich weniger Zeit mit Freund*innen, Verwandten oder Nachbar*innen und erleben doppelt so häufig Gefühle von Einsamkeit als Menschen ohne Beeinträchtigungen [7].
- *Häufigere Erfahrungen sexualisierter Gewalt*: Von sexualisierter Gewalt sind Menschen mit Behinderung grundsätzlich stärker betroffen als Menschen ohne Behinderung. Besonders gefährdet sind Frauen [26] sowie Menschen, die in Institutionen der EGH leben [27].
- *Ggf. zusätzliche körperliche Herausforderungen*: Menschen mit körperlichen Beeinträchtigungen stehen je nach Ausprägung ihrer individuellen körperlichen Situation vor der Herausforderung, Hilfsmittel oder Assistenz zur Realisierung ihrer Sexualität arrangieren zu müssen. Diese kann neben der finanziellen Belastung auch an normativen Vorstellungen der Fachkräfte scheitern oder aber die Institutionen begreifen dies nicht als Teil ihres fachlichen Auftrags. In Deutschland kann – trotz einiger juristischer Einzelfallentscheidungen – Sexualassistenz immer noch nicht als Teil von Teilhabeleistungen finanziert werden.
- *Sprachlosigkeit – geringe Erfahrung sexueller Bildung*: Menschen mit Behinderung erfahren in ihrem Leben häufig weniger Angebote sexueller Bildung als andere Menschen [6]: Zusammen mit geringer Kenntnis sexueller Rechte können reduzierte oder fehlende Sprach- und Sprechkompetenzen zu einer großen Sprachlosigkeit in Bezug auf sexuelle Themen führen. Dies wiederum erhöht die Vulnerabilität für sexuelle Gewalt und erschwert die Kommunikation individueller (sexueller) Wünsche.

Bei der Skizzierung dieser Faktoren gilt es zu beachten, dass es sich bei Fragen sexueller Selbstbestimmung nicht um ein zu vernachlässigendes Randthema, sondern um einen zentralen Lebensbereich mit Interdependenzen zu relevanten weiteren Themen der Lebensführung und Gesundheit handelt.

## Das Projekt ReWiKs

**Abb. 1:**
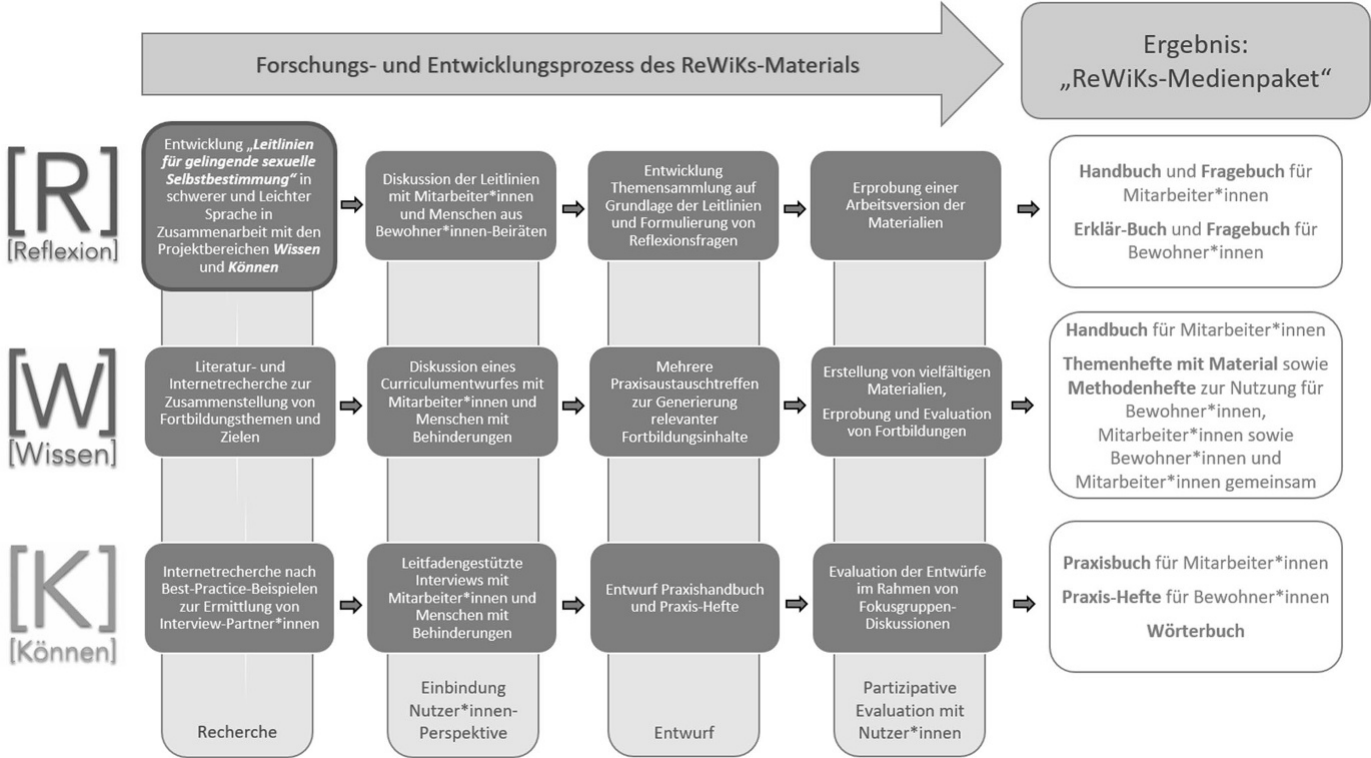
Forschungs- und Entwicklungsprozess des ReWiKs-Materials

Vor dem oben skizzierten Hintergrund startete im November 2014 das Projekt „ReWiKs“ in einer ersten Förderphase an 3 Hochschulstandorten (Bochum, Münster, Landau/später Berlin; [2]). Bis 05/2019 wurden umfangreiche Materialien in leichter Sprache und in Alltagssprache entwickelt (Abb. 1.). Das entstandene „ReWiKs-Medienpaket“ [8] besteht aus insgesamt 36 Publikationen, die sich auf die 3 Bereiche „Reflexion“, „Wissen“ und „Können“ aufsplitten. Neben der Materialentwicklung wurden Fortbildungen für Mitarbeitende entwickelt und evaluiert. Für Assistenznehmer*innen wurden Austauschformate („Arbeitskreise“) entwickelt und erprobt. Die Formate dienten der themenspezifischen Auseinandersetzung mit Haltungen, Strukturen und Praktiken in den Einrichtungen und Diensten der EGH und der Weiterbildung beider Zielgruppen.

### Das ReWiKs-Medienpaket

Das Material im Bereich „Reflexion“ dient der Auseinandersetzung mit Haltungen, Strukturen und Praktiken in Bezug auf sexuelle Selbstbestimmung und zur systematischen Erkundung von Veränderungsbedarfen in den Einrichtungen und Diensten der EGH (Fragenkataloge in Alltagssprache sowie in Leichter Sprache).

Das Material im Bereich „Wissen“ nimmt sich der Erweiterung des organisationalen Wissens zum Themenfeld Sexualität und Behinderung an („Themenhefte“ mit Anregungen zur Auseinandersetzung in Alltagssprache). Zum Bereich Wissen zählt auch ein Materialfundus, der aus 200 Materialien in Alltagssprache und leichter Sprache besteht („Leitlinien gelingender sexueller Selbstbestimmung“, Fortbildungsentwürfe, Arbeitshilfen für die Praxis u. v. m.).

Das Material des Bereichs „Können“ stellt Informationen in Alltagssprache und in Leichter Sprache bereit (Nachschlagewerke zu Begriffen und Konzepten sowie Handlungsempfehlungen und Informationen zu Einzelthemen).

### Zweite Förderphase – Bundesweite Verbreitung

**Abb. 2:**
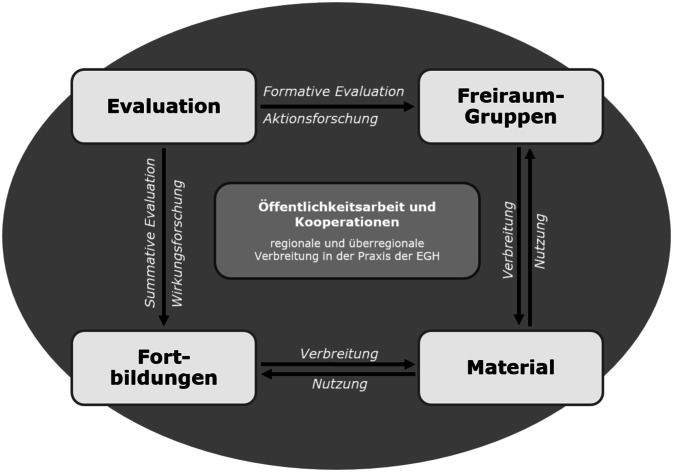
Vorgehen in der zweiten Förderphase des Projekts ReWiKs, Ziele der Projektbausteine. *EGH* Eingliederungshilfe

Die 2. Förderphase des Projektes von 06/2019 bis 12/2023 („Sexuelle Selbstbestimmung und Behinderung – Reflexion, Wissen, Können als Bausteine für Veränderungen“) wurde an der Katholischen Hochschule Nordrhein-Westfalen (Standort Münster) und an der Humboldt-Universität zu Berlin durchgeführt und hatte das Ziel, das ReWiKs-Medienpaket sowie die Weiterbildungs- und Kommunikationsformate bundesweit und nachhaltig in Einrichtungen und Diensten der EGH zu verbreiten (Abb. 2). Die Fortbildungsveranstaltungen „ReWiKs-Lots*innen“ für Fachpersonal wurden an verschiedenen Standorten sowie bundesweit angeboten. Zusätzlich wurden Vernetzungsformate für den nachfolgenden Austausch geschaffen. Die „Arbeitskreise“ für Assistenznehmer*innen^1^ wurden in partizipationsorientierte Kommunikationsformate, sog. Freiraum-Gruppen, weiterentwickelt und in Kooperation mit Selbstvertretungsorganisationen angeboten. Sogenannte Freiraum-Begleiter*innen erwarben Kompetenzen zur Organisation der Gruppen. Materialschulungen zum „ReWiKs-Medienpaket“ wurden bundesweit angeboten, um den Einstieg in die Arbeit mit der Materialsammlung zu erleichtern und so zu einer nachhaltigen Nutzung des Medienpakets beizutragen.

Für eine bundesweite Sichtbarkeit in der Fachöffentlichkeit setzte das Projekt eine breit angelegte Kommunikations- und Kooperationsstrategie um. Auf regionaler Ebene kooperierte es mit Fachverbänden und Selbstvertretungsorganisationen, führte Veranstaltungen und Projektpräsentationen durch und nutzte soziale Medien (Instagram) zur Öffentlichkeitsarbeit. Die Ergebnisse wurden der Fachöffentlichkeit in 2 Abschlussveranstaltungen sowie diversen Materialien und Fachpublikationen präsentiert, zuletzt in einem eigenen Sammelband [4].

Die 2. Förderphase wurde durch eine Programmevaluation begleitet, die sich der Beschreibung und Untersuchung der Bedingungen, Konzeptionen, Aktivitäten und Resultate widmete [28]. Die Evaluation der Lots*innen-Fortbildungen zielte auf die Untersuchung der Wirkung der Fortbildung im Sinne eines Theorie-Praxis-Transfers sowie des Rollenverständnisses der Fortbildungsteilnehmenden. Die Evaluation der Freiraum-Gruppen untersuchte die Herausforderungen und Gelingensbedingungen ihrer Implementierung in der Praxis der Selbsthilfe, EGH und Beratung sowie individuelle Wirkungen bei den teilnehmenden Menschen mit Behinderungen.

## Die ReWiKs-Ergebnisse – zur Wirkung der Aktivitäten

### Ergebnisse zur Materialentwicklung und zur Verbreitung des Materials

Erkenntnisse zur Nutzung und Akzeptanz des Medienpakets wurden aus verschiedenen Datenquellen zusammengeführt (Befragung der Fortbildungsteilnehmenden, Protokolle, Rückmeldungen aus Veranstaltungen). Das Medienpaket wurde überwiegend positiv bewertet. Der Umfang und die Vielfältigkeit des Materials wurden von Fachpersonen als gewinnbringend, zugleich auch als Herausforderung wahrgenommen. Aus Sicht der Nutzer*innen bedarf es für den Einstieg in die Arbeit mit dem Medienpaket einer strukturierten Einführung. In Konsequenz werden durch das BIÖG Materialschulungen in Leichter Sprache und in Alltagssprache angeboten.^2^ Die Qualitätsbewertung der 36 Materialien war positiv, insbesondere die Materialien in Leichter Sprache wurden als sehr gelungen bewertet. Das Medienpaket wurde am häufigsten zur Informationsvermittlung, Beratung und Aufklärung verwendet sowie für Fortbildungen und in der alltäglichen Begleitung von Assistenznehmer*innen eingesetzt.

### Ergebnisse aus der Lots*innenfortbildung und Vernetzung von Fachpersonal

Insgesamt nahmen an den 13 Fortbildungsdurchläufen 280 Personen aus 183 Einrichtungen und Diensten der EGH, Beratungs- sowie Selbsthilfeeinrichtungen teil (erfolgreicher Abschluss mit Zertifikat *n* = 203). Die Fortbildungsbildungsdurchläufe hatten einen regionalen Fokus, um nachhaltige Impulse vor Ort zu setzen. Jeweils ein Durchlauf wurde in Baden-Württemberg, Bayern, Hamburg, Hessen, Mecklenburg-Vorpommern, Sachsen, Sachsen-Anhalt, Thüringen sowie jeweils 2 Durchläufe in Nordrhein-Westfalen und Berlin-Brandenburg angeboten. Aufgrund der großen Nachfrage wurde zusätzlich ein bundesweiter Kurs angeboten.

In 5 Regionen wurde mit den „ReWiKs-Foren“ eine kontinuierliche Vernetzung der Fortbildungsteilnehmenden etabliert. Zusätzlich wurden digitale Vernetzungsangebote mit wechselnden thematischen Schwerpunkten eingeführt, sogenannte offene Web-Seminare, die bis heute bestehen.^3^

Die Fortbildung wurde mit einem Mixed-Methods-Ansatz in Form eines sequenziellen Vertiefungsdesigns evaluiert [29]. Der quantitative Studienteil wurde als Ein-Gruppen-Prä-Post-Design mit Follow-up umgesetzt (t0 = Baseline, t1 = Ende der Fortbildung, t2 = 6 Monate nach Fortbildung), ergänzt durch einen qualitativen Studienteil (11 Interviews).

Von den 246 Personen, die an mindestens einer der 3 Befragungen teilnahmen, waren 64 % (*n* = 157) in Organisationen der EGH im Bereich „Wohnen“, 15 % (*n* = 40) als Beratungspersonen und 18 % (*n* = 49) als Mitarbeitende in sonstigen Einrichtungen (z. B. Werkstätten für behinderte Menschen, Selbsthilfe) tätig. Die gemischte Zusammensetzung der Fortbildungsgruppen wurde als gewinnbringend bezeichnet.

Präsentiert werden die Ergebnisse der Befragung der Mitarbeitenden im Bereich „Wohnen“.

#### Strukturen, Haltungen und Praktiken in den Einrichtungen und Diensten

56 % der Befragten gaben zu t0 an, mindestens einmal wöchentlich mit sexuellen Themen konfrontiert zu sein. 56 % bezeichneten das Thema als „bedeutsam“ oder „sehr bedeutsam“. Eine organisationale Verankerung in Form sexualpädagogischer Konzepte oder im Leitbild wurde nur von einem Viertel der Befragten angegeben, beim Rest war diese entweder nicht vorhanden oder nicht bekannt. Es besteht eine Diskrepanz zwischen der Bedeutung des Themas in den Einrichtungen und der organisationalen Verankerung, was ein potenzielles Spannungsfeld anzeigt.

Dass sexuelle Selbstbestimmung in der Organisation häufiger Thema sein sollte, fanden 56 % der Befragten. Hauptgründe für eine erschwerte Thematisierung sexueller Selbstbestimmung zu t0 liegen in unzureichenden fachlichen Kenntnissen und Handlungskompetenzen der Kolleg*innen sowie mangelnden zeitlichen und personellen Ressourcen, was sich auch zu t2 nicht verändert hat. Seltener wurde eine fehlende Offenheit der Kolleg*innen oder der Leitung als Erschwernis angegeben. Es bestehen ein Interesse auf Einrichtungsseite und Seite der Klient*innen sowie eine grundsätzliche Offenheit der Einrichtung für Veränderungen in diesem Bereich [30].

Zum Umgang mit Wünschen, Bedürfnissen und Vorstellungen der Klient*innen gab lediglich ein Viertel der Befragten zu t0 an, die Wünsche und Bedarfe der Klient*innen bezogen auf sexuelle Selbstbestimmung aktiv zu ermitteln. Information und Beratung der Klient*innen sowie deren Dokumentation gab jeweils rund die Hälfte der Befragten als organisationale Praktiken an (Kategorie „trifft eher zu“: 48–58 %). Über 40 % der befragten Lots*innen gaben an, dass keine Weitervermittlung von Klient*innen an externe Beratungsstellen stattfindet. Nur ein knappes Drittel der Befragten bestätigte themenspezifische Kooperationen mit externen Akteur*innen bzw. Organisationen (regelmäßiger Austausch nur 12 %).

#### Ausgestaltung der Rolle und Funktion der Lots*innen

Der Theorie-Praxis-Transfer der Lots*innen-Fortbildung gelingt vor allem hinsichtlich der fachlichen Kompetenzen der Mitarbeitenden. Diese geben zu t1 deutliche Wissenszuwächse im Bereich der Konzept- und Organisationsentwicklung sowie der Kompetenzen zur Erweiterung der sexuellen Selbstbestimmung der Klient*innen an, die auch zu t2 sichtbar blieben [30]. Organisationale Rahmenbedingungen veränderten sich im Beobachtungszeitraum kaum. Die Offenheit der Einrichtung für das Thema sowie die Einschätzung, inwiefern sexuelle Selbstbestimmung dort umgesetzt werden kann, nahmen zu t1 leicht zu. Durch Impulse der Lots*innen wurden in relevantem Maße Arbeitskreise, sexualpädagogische Konzepte oder die Arbeit an Leitbildern weiterentwickelt oder neu initiiert [30]. Strukturelle Veränderungen im Sinne einer sexualitätsfreundlichen Organisationsentwicklung sind im Projekt- und Evaluationszeitraum nicht erkennbar. Als Multiplikator*innen bringen die ReWiKs-Lots*innen das Thema jedoch in ihren Organisationen ein und geben ihr Wissen an Mitarbeitende und Klient*innen weiter.

### Effekte der Freiraum-Gruppen

Die Freiraum-Gruppen sind ein Begegnungsformat für erwachsene Menschen mit Lernschwierigkeiten, das in einem geschützten Raum (meist außerhalb von Einrichtungen der EGH) einen Austausch zu den Themen Liebe, Partnerschaft, Sexualität und Selbstbestimmung ermöglicht. In den Gruppen geht es primär um die selbstbestimmte Auseinandersetzung der Teilnehmenden mit Bedürfnissen, Gefühlen und Fragen im Kontext von Sexualität und Selbstbestimmung [31].

In intensiver Zusammenarbeit mit Selbstvertretungsorganisationen wurden die Gruppen implementiert und mit einem an die Aktionsforschung angelehnten teilpartizipativ orientierten Design formativ evaluiert. Ziel war es, Herausforderungen und Gelingensbedingungen der Implementierung herauszuarbeiten sowie Wirkungen der Gruppen auf individueller Ebene der Teilnehmenden nachzuzeichnen. Selbstvertreter*innen wurden als sog. Freiraum-Begleiter*innen geschult, die die Gruppen organisierten. Im Sinne der Aktionsforschung wurden diese zudem in die wissenschaftliche Begleitung einbezogen (Feldnotizen, Beteiligung an Reflexionsformaten). Eine Partizipation von Gruppen-Teilnehmenden an der Forschung konnte in einzelnen Phasen umgesetzt werden (z. B. Auswertungsworkshop [32]).

In die Datenauswertung gingen 7 vom Projekt begleitete Gruppen ein. Im Zeitraum 2020–2023 wurden 275 Freiraum-Gruppen-Termine durchgeführt. Die 7 Gruppen erreichten 62 Assistenznehmer*innen der EGH. Es wurden 32 Personen mit und ohne Behinderungserfahrungen als Freiraum-Begleiter*innen geschult.

Herausforderungen und Gelingensbedingungen der Implementierung und Durchführung der Gruppen können dem Evaluationsbericht des Projekts entnommen werden [30]. Die nachfolgende Darstellung fokussiert auf die Effekte der Freiraum-Gruppen auf individueller und Gruppenebene [33].

Die Gruppen wirkten sich auf individueller Ebene persönlichkeitsstärkend, wissens- und kompetenzerweiternd aus, was eindrücklich mit längsschnittlichen Beobachtungen dokumentiert wurde. Teilnehmende gingen gestärkt aus den Gruppenprozessen hervor, Empowerment-Prozesse wurden unterstützt. Es konnte herausgestellt werden, dass die Gestaltungsprinzipien der Gruppen, vor allem die partizipative Ausrichtung und die Aspekte des Peer Counseling, Peer Learning und Peer Support, sowie ein Wissenszuwachs bei den Teilnehmenden wesentlich zu dieser Wirkung beitrugen [33]. Das Erleben von Resonanz auf die eigenen Äußerungen in einem geschützten Raum sowie die Bestärkung durch die Freiraum-Begleiter*innen und Forscher*innen, die im Sinne der Aktionsforschung auch Teil der Gruppen waren, unterstützten das Empowerment der Teilnehmenden. Sie gestalteten einen Raum ohne einstellungsbedingte Barrieren in Bezug auf ihr Recht auf sexuelle Selbstbestimmung. Ihre Äußerungen wurde anerkannt und sie wurden als „sexual beings“ wahrgenommen [34]. Auf der anderen Seite zeigte sich, dass die Freiraum-Gruppen Routinen in den Organisationen der EGH störten und durch das Hinterfragen eingespielter Abläufe für Unruhe sorgten. So wurden fremdbestimmende Strukturen und Praktiken sowie Handlungsoptionen für mehr Selbstbestimmung im Rahmen der Freiraum-Gruppen aufgezeigt und Teilnehmende bestärkt, für ihre Rechte einzustehen.

## Fazit – Sexuelle Selbstbestimmung: auf einem guten Weg?

Sexuelle Selbstbestimmung von Menschen mit Behinderung lässt sich angemessen verstehen und realisieren, wenn Sexualität als vielschichtiges, menschenrechtlich gerahmtes Feld betrachtet wird, das rechtliche, politische, institutionelle, diskursive und individuelle Dimensionen umfasst. In bestehenden Diskursen zeigt sich, dass Behinderung in sexualitätsbezogenen Kontexten häufig individualisiert, entsexualisiert oder einseitig resexualisiert wird, während Fragen von Geschlecht, Queerness und Intersektionalität marginal bleiben [35, 36]. Notwendig sind interdisziplinäre Perspektiven, die sonder- und rehabilitationspädagogische, soziologische, juristische und Disability-theoretische Ansätze verbinden und Sexualität nicht als Risiko, sondern als Bereich von Rechten, Ressourcen und Teilhabe betrachten [37, 38].

Im *politisch-gesellschaftlichen Raum* wird die Bedeutung des Themas besonders dort sichtbar, wo konkrete Aktionspläne und Förderinstrumente die Lebenslagen von Menschen mit Behinderung adressieren (z. B. LSBTIQ+ [39]). Solche Maßnahmen tragen dazu bei, sexuelle Themen öffentlich zu verankern und Wege sexueller Teilhabe zu eröffnen, zeigen aber zugleich, dass politische Programme mit strukturellen Veränderungen in Einrichtungen und Diensten verzahnt werden müssen.

Im Feld der *sexuellen Bildung* zeigt sich, dass Menschen mit Lernschwierigkeiten und anderen Beeinträchtigungen auf leicht zugängliche, empowernde Angebote angewiesen sind, um Wissen über Körper, Beziehungen, Rechte, Gewaltprävention und eigene Wünsche aufzubauen [40, 41]. Praxisprojekte, Weiterbildungen für Fachkräfte in der EGH, Freiraum-Gruppen in Kooperation mit Selbstvertretungen sowie das ReWiKs-Medienpaket zielen darauf ab, sexualfreundliche Institutionen zu etablieren. Bislang integrieren Ausbildungs- und Studiengänge inklusive sexualitätsspezifische Inhalte oft nicht in ihre Curricula. Schulische sexuelle Bildung für Kinder und Jugendliche mit Behinderung bleibt somit unterrepräsentiert und führt trotz zum Teil adäquater Bildungsmaterialien [8, 42] zu Aufklärungs- und in der Folge zu Selbstbestimmungsdefiziten.

Auf der Ebene *rechtlicher Rahmungen und Teilhabeplanung* wird deutlich, dass das Recht auf sexuelle Selbstbestimmung zwar aus allgemeinen Selbstbestimmungsrechten ableitbar ist, jedoch im Bundesteilhabegesetz (BTHG § 4 (1)) und Teilhabeberichten selten explizit Erwähnung findet [43]. Ansätze, sexualitätsbezogene Bedürfnisse über spezifische ICF-Codes zu codieren, eröffnen die Möglichkeit, sexualitätsbezogene Assistenzleistungen^4^ als Teilhabeleistungen zu begründen [44–47] und letztlich auch als solche zu finanzieren. Spannungsfelder zwischen Privatsphäre und Offenlegung erfordern, dass sensible Verfahren wie verschlüsselte Thematisierungen oder Vorabgespräche Einzug in Teilhabeverfahren halten [48, 49].

Für die *Weiterentwicklung von Wohneinrichtungen* hin zu sexualfreundlichen, antiableistischen Institutionen ist ein breites Kompetenzprofil von Fachkräften erforderlich, dass Reflexionsfähigkeit, gendersensible Assistenz, Wissen zu Rechten und Präventionskompetenzen umfasst [8]. Die im Projekt ReWiKs entwickelten Leitlinien gelingender sexueller Selbstbestimmung können als Orientierungsrahmen dienen, um partizipative Prozesse mit Bewohner*innen anzustoßen und Organisationsentwicklung an deren Perspektiven auszurichten. Menschen mit Behinderung agieren dabei als Expert*innen ihrer eigenen Sexualität, deren Entscheidungen im Zentrum von Recht, Politik und Praxis stehen müssen.

## Data Availability

Alle dieser Arbeit zugrunde liegenden Daten sind in diesem Artikel enthalten.

## Footnotes

1 Die Assistenznehmer*innen wurden über Einrichtungen der EGH sowie Organisationen der Selbstvertretung akquiriert.

2 https://www.sexualaufklaerung.de/qualifizierung/rewiks/. Zugegriffen 05.03.2026.

3 https://www.sexualaufklaerung.de/qualifizierung/rewiks/offene-rewiks-foren-digital. Zugegriffen 05.03.2026.

4 Unter Assistenzleistungen werden verschiedene Formen aktiver oder passiver Sexualassistenz bzw. Sexualbegleitung subsumiert, die Menschen mit einem spezifischen Unterstützungsbedarf in Anspruch nehmen können, um ihre Sexualität leben zu können (vgl. vertiefend [50]).
